# Association between Emotional Intelligence and Professionalism in Medical Students: The Compassion-Competence Nexus

**DOI:** 10.12669/pjms.41.1.9748

**Published:** 2025-01

**Authors:** Javeria Rehman, Syed Imran Mehmood

**Affiliations:** 1 Javeria Rehman, MBBS, MHPE Department for Educational Development, Department of Pathology and Laboratory Medicine, The Aga Khan University, Karachi, Pakistan; 2 Syed Imran Mehmood, MBBS, MA, MMedED (UK), PhD (Netherlands) Dow Institute of Health Professionals Education, Dow University of Health Sciences, Karachi, Pakistan

**Keywords:** Emotional intelligence, Professionalism, TEIQue-SF, Modified PMEX

## Abstract

**Background and Objective:**

In medical education, the challenging constructs of emotional intelligence and professionalism are increasingly being addressed worldwide and seem to share common characteristic components. The objective of this study was to determine the association between emotional intelligence and professionalism as perceived and self-reported by medical students and to explore the gender difference in these two variables.

**Methods:**

It is a cross-sectional study of eight months duration, from February-September 2019, that included final year medical students at Dow Medical College through convenience sampling. The participants completed the self-reporting questionnaires of trait emotional intelligence and modified professionalism mini evaluation exercise. Pearson correlation coefficient was applied to determine the association between the emotional intelligence and professionalism scores. Independent T test was used to determine the gender difference for these two variables.

**Results:**

Emotional Intelligence and professionalism scores were found to be positively and significantly correlated with moderate strength of association (r =0.412). There was no significant difference of emotional intelligence and professionalism scores of males and females.

**Conclusions:**

Increase in emotional intelligence is associated with the increase in professionalism among medical students.

## INTRODUCTION

Medical profession is pivoted on human interactions. Medical education aspires to promote and nurture attributes in physicians that would enable them to provide high quality of patient centered care.^1^ Research suggests that such attributes, which may manifest as personality traits or skills, are also components of emotional intelligence (EI) and require their appropriate management and demonstration to achieve professional competence of a “smart and good” doctor.^2^,^3^ In medical education, the challenging constructs of EI and professionalism are increasingly being addressed and inquired worldwide.^3^ “Emotional Intelligence” deals with the management of one’s own emotions as well as the emotions of others.^4^ Researchers have proposed different concepts and definitions of EI. As presented by Petrides et al (2016), EI can be termed as a “trait”, where it deals with one’s perception of one’s emotional abilities concerned with his/her understanding, regulation and expression of emotions.^5^ The trait concept is based on “emotional self-efficacy” and includes the domains of adaptability, assertiveness, emotion perception (self and others), expression, management and regulation, self-esteem, self-motivation, social awareness, stress management, trait empathy, happiness and optimism.^5^ Research in cognitive and neurosciences suggests identification, perception and interpretation of information that leads to the ultimate action by the healthcare professionals is greatly influenced by their emotions.^6^

Professionalism is demonstrated by various responsibilities such as clinical competence, scientific knowledge, effective communication skills and comprehensive conception of legal and ethical practice.^7^ As suggested by various studies, such as those by Arora et al (2010), Cherry et al (2012), Cherry et al (2014) and Johnson (2015), it is anticipated that health care professionals who communicate better and demonstrate higher levels of professionalism are more emotionally intelligent.^1^,^3^,^6^,^8^ Lack of emotional well-being is associated with physician’s stress, burnout, ineffective communication, lack of empathy, patients’ dissatisfaction, conflicts and poor doctor patient relationship, which eventually results in disapproval towards physician’s professionalism and may even cause an increasing mistrust and violence against the healthcare professionals.^9^,^10^

Despite the requisite of professional qualities in a medical graduates mentioned in undergraduate curriculum and as stated by accreditation bodies locally and worldwide, unprofessional behaviours have been increasingly observed not only in medical education but also in medical practice.^11^ Both EI and professionalism of medical students remain relatively ignored concepts in Pakistan.^12^,^13^ Investigating association between emotional intelligence and professionalism of medical students will identify the need of addressing the issue formally in the form of required and targeted curricular interventions. To the best of our knowledge no study in local context has identified the link between EI and professionalism in undergraduate medical students of Pakistan. Hence, the purpose of the study was to assess and determine association between EI and professionalism in undergraduate medical students of final year in a public sector medical college in Karachi.

## METHODS

The cross-sectional study was conducted at Dow Medical College Karachi in a duration of eight months February-September 2019. The study population included a universal sample of all 350 medical students from final year, through convenience sampling. The study parameters mainly included the Global trait EI score and the professionalism score as measured by self-reporting trait emotional intelligence questionnaire - short form (TEIQue-SF) and modified professionalism mini evaluation exercise (PMEX) questionnaire, respectively. Other variables included age and gender of the participants.

### Ethical approval:

It was obtained from the Institutional Review Board (IRB) of Dow University of Health Sciences (Ref: IRB 1157/DUHS/Approval/2018; Date: November 22,2018).

After obtaining informed consent, the study participants were invited to complete the TEIQue-SF and modified PMEX questionnaires. Data was collected anonymously, and the responses were collected immediately after completion of questionnaires. The data was secured to maintain the confidentiality and anonymity of the participants.

### Instruments:

### TEIQue-SF:

The 30-item TEIQue-SF was designed to efficiently measure global trait EI based on four trait EI factors namely ‘well-being,’ ‘self-control,’ ‘emotionality,’ ‘sociability,’. A Likert scale ranging from one (completely disagree) to seven (completely agree) is used for the responses to the test items. The TEIQue-SF is a self-reporting questionnaire adapted from the full form of the TEIQue, which contains four factors with 15 subscales and measures the behaviour tendencies in a variety of situations.^14^ Two items from each subscale were selected for the short version.^5^ The subscales of adaptability and self-motivation contribute directly to the global trait EI.^5^ The factors are well-being, self-control, emotionality and sociability.^5^ TEIQue-SF is available freely for academic research purposes and has good psychometric properties.^14^

### PMEX Questionnaire :

The modified PMEX- Questionnaire is a self-reporting 20-item instrument based on the PMEX originally designed to be used in objective assessment of student’s behaviour related to professionalism.^15^ The participants perceived and self-reported their own professional behaviour related to each item on a Likert scale ranging from “never=1 ” to “always=5”. The modification also included changing of words or splitting the item into two for the ease of understanding and to best apply in local context of participants. The permission of modifying and using the instrument was taken from the authors and the modified version was reviewed and approved by the authors of original PMEX and was also pilot tested.

### Statistical Analysis:

The data was analyzed by using IBM Statistical Package for the Social Sciences (SPSS) 21. Mean with SD for age and scores of EI and professionalism were determined as part of descriptive statistics. Test for Pearson correlation coefficient was applied to determine correlation between EI and professionalism, and also between professionalism and the four factors of the trait EI namely well-being, self-control, emotionality and sociability. The mean scores of EI and professionalism of male and female students were compared by using independent t-test with statistical significance taken as p-value of <0.05. The internal consistency of both the instruments was determined by Cronbach’s alpha (α) in this study.

## RESULTS

A total of 204 (N= 204) students participated in the study with a response rate of 58.2%. Participants included 152 (74.51%) females whereas 52 (25.49%) were males. The mean age ± standard deviation (SD) of the participants was 22.58 ± 0.74 years. Table-I shows the mean professionalism score and mean Global score of trait EI of the participants. The participants scored highest in well-being while scored lowest in sociability as shown in Table-I.

**Table-I:** Mean professionalism and EI scores.

	Minimum	Maximum	Mean±SD
Professionalism score	2.50	5.00	3.92±0.5
Global trait EI score	2.67	6.07	4.37±0.7
***Factors of TEIQue-SF***			
Well-being	2.0	7.0	4.82±1.12
Self-control	1.17	6.33	4.09±1.0
Emotionality	2.75	6.63	4.49±0.85
Sociability	1.20	6.33	4.08±0.95

The difference between the mean scores of professionalism as well as EI of both genders was found to be statistically insignificant as summarized in Table-II.

**Table-II:** Difference in mean professionalism and EI scores of male and female medical students.

	Professionalism score Mean ± SD	Global trait EI score Mean ± SD	Factor scores of TEIQue-SF Mean ± SD			

			Well-being	Self-control	Emotionality	Sociability
Male (n=52)	3.85 ± 0.49	4.38 ± 0.70	4.91 ± 1.18	4.01 ± 1.11	4.52 ± 0.94	4.07 ± 0.98
Female (n=152)	3.95 ± 0.51	4.37 ± 0.70	4.79 ± 1.10	4.12 ± 0.97	4.49 ± 0.83	4.09 ± 0.95
p-value	0.25	0.93	0.52	0.51	0.84	0.91

The increase in EI scores showed an increase in professionalism scores of the participants as there was positive association between the scores of Global trait EI and professionalism. The strength of this association was moderate and was found to be statistically significant with a p-value of <0.0001. Correlations between professionalism and four factors of trait emotional intelligence were also determined as shown in Table-III.

**Table-III:** Correlation between Professionalism and EI with its four factors.

	Professionalism	p-value
Global trait EI	0.412*	<0.0001
Well-being	0.328*	<0.0001
Self-control	0.281*	<0.0001
Emotionality	0.264*	<0.0001
Sociability	0.231*	0.001

* . Correlation is significant at p<0.01 level.

Reliability of the instruments with Cronbach’s α was found to be 0.80 for TEIQue-SF and 0.88 for modified PMEX.

## DISCUSSION

This study provides evidence of a moderate, positive, and significant association between EI and professionalism, especially in our local context, with a correlation coefficient (r)=0.412. To the best of our knowledge no study in local context has investigated the relationship between EI and professionalism in undergraduate medical students of Pakistan. Literature indicates that a physician’s emotional intelligence (EI) is a crucial factor, encompassing various attributes that contribute to a competent physician.^1^,^3^,^6^,^8^ However, there is dearth of literature regarding association of EI with comprehensive or holistic measure of professionalism using a professionalism measuring instrument.^16^ Therefore, very limited studies in local or international context were available to compare the findings.

The mean professionalism scores of this study’s participants was found to be somewhat moderate as 3.92±0.5, but higher than those reported by Karukivi et al. in Finnish medical students using PMEX.^17^ Similar but slightly higher professionalism scores were reported in dental students in United States.^18^ This study’s finding is suggestive of similarity with local studies that reported inadequate and suboptimal professionalism levels in undergraduate medical students.^19^,^20^

The mean Global EI score of 4.37±0.7 in this study participants can be considered comparable to the values of EI reported in other studies in undergraduate medical students of Pakistan.^21^ Similar but slightly higher values of EI have been reported in medical students of Japan and Iran.^22^,^23^ Furthermore, the participants of the present study scored highest in ‘well-being’ factor and lowest on ‘sociability’ factor of EI which is in similarity with the findings of various local and international studies.^24^,^25^ The finding of present study is in agreement with McNulty et al (2016) who reported lower values for EI in Asian students as compared to the western world. This could be due to the reason that students in Asian culture tend to underrate their intelligence when self-reporting, as mentioned by other authors.^25^

In this study, the statistically insignificant difference of professionalism scores (p-value=0.25) among male and female participants is in consistency with various studies which also reported no significant difference in professionalism scores on the basis of gender.^17^,^19^,^26^,^27^ This study also reported comparable EI scores between male and female medical students with statistically insignificant difference (p-value=0.93), which aligns with similar findings in literature determining no gender difference in EI among medical students.^16^,^21^-^23^,^28^,^29^ Contrasting results were reported in other studies where females scored significantly higher in EI as compared to males, while in some studies it was reported vice versa.^24^,^25^,^30^,^31^

Similarly, there was no statistically significant difference in all the four factors of EI among genders. This is in agreement to the findings of Mahaur et al (2017) who reported no significant difference in the four EI factors among males and females.^29^ A different result was reported by Ahmed et al (2017) where male medical students were significantly high scorers in all four factors of EI.^24^ In this study, the overall similar mean scores of EI and professionalism in both the genders can be explained due to exposure to a similar educational and social environment.

The present study found a positive, significant relationship between the mean scores of EI and professionalism in partial concordance with that of Farah-Franco (2017) who found positive but statistically insignificant relationship between the two variables.^18^ Study by Partido et al (2021) on dental students also reports similar findings as moderate but statistically significant association between the total EI and professionalism scores as well as between EI domains with total professionalism scores.^16^ Similar to this study, several studies have reported positive relationship between EI and many aspects of professionalism including doctor patient relationship, interpersonal skill and teamwork.^28^ Reed et al (2015) and Humphrey-Murto et al (2014) reported contradicting findings suggesting no correlation of EI with certain aspects of professionalism.^32^,^33^

This study also determined the reliability of both the instruments that was found to be 0.8 which is excellent and in close proximity to other previously reported studies.^14^,^18^ Due to extensive relevance of EI to many aspects of professional competencies in medicine, there has been a worldwide rise in the demand for incorporating such elements in undergraduate as well as post graduate medical education with formal teaching and assessment methods.^28^ Investigating the association between emotional intelligence and professionalism of medical students, this study has provided insight into the EI and professionalism levels of medical students. Findings have revealed the need to address the issue formally in the form of required and targeted curricular interventions to improve EI and professionalism. However, development and implementation of curricular reforms regarding professionalism and EI must be in alignment with one’s own socio-cultural context.

### Limitations:

This study has certain limitations. It was a cross-sectional study using self-reported questionnaires, hence does not imply any cause-and-effect relationship between emotional intelligence and professionalism. The study was conducted on a single cohort at a single institution with convenience sampling therefore the findings cannot be generalized. Moreover, very few similar studies in local or international context were available to compare the findings. Future research could benefit from a multicenter and multisource assessment that could provide further insights into the true performance and competence of medical students.

## CONCLUSION

There was a moderate, positive and significant association between EI and professionalism scores of the study participants which suggests that increase in EI is associated with the increase in professionalism. The male and female students were found to be similar in their EI and professionalism. Interventions and specialized training programs aimed at enhancement of EI in medical students can be developed and investigated as experimental and longitudinal studies to determine their effect on professionalism.

## References

[1] Arora S, Ashrafian H, Davis R, Athanasiou T, Darzi A, Sevdalis N (2010). Emotional intelligence in medicine:a systematic review through the context of the ACGME competencies. Med Educ.

[2] Chew BH, Zain AM, Hassan F (2013). Emotional intelligence and academic performance in first and final year medical students:a cross-sectional study. BMC Med Educ.

[3] Cherry MG, Fletcher I, O'sullivan H, Shaw N (2012). What impact do structured educational sessions to increase emotional intelligence have on medical students?BEME Guide No. 17. Med Teach.

[4] Mayer JD, Sluyter DJ (1997). What is emotional intelligence?P Salovey. Emotional Development and Emotional Intelligence.

[5] Petrides KV, Mikolajczak M, Mavroveli S, Sanchez-Ruiz M-J, Furnham A, Pérez-González J-C (2016). Developments in trait emotional intelligence research. Emot Rev.

[6] Cherry MG, Fletcher I, O'Sullivan H, Dornan T (2014). Emotional intelligence in medical education:a critical review. Med Educ.

[7] Birden H, Glass N, Wilson I, Harrison M, Usherwood T, Nass D (2014). Defining professionalism in medical education:a systematic review. Med Teach.

[8] Johnson DR (2015). Emotional intelligence as a crucial component to medical education. Int J Med Educ.

[9] Blanchard C, Kravets V, Schenker M, Moore Jr T (2021). Emotional intelligence, burnout, and professional fulfillment in clinical year medical students. Med Teach.

[10] Sundararajan S, Gopichandran V (2018). Emotional intelligence among medical students:a mixed methods study from Chennai, India. BMC Med Educ.

[11] Khan JS, Tabasum S (2011). Medical professionalism:a panoramic view through the kaleidoscope of stakeholder perspectives. J Ayub Med Coll Abbottabad.

[12] Mughal AM (2022). Professionalism-The Ignored Competence of Medical Education in Pakistan. Pak J Med Dent.

[13] Chaudhry MA, Rafeeq S, Khan DA, Nosheen J, Munir S (2023). Prevalence of Emotional Intelligence in Students at a Medical College in Pakistan. Ann King Edw Med Univ.

[14] O'Connor PJ, Hill A, Kaya M, Martin B (2019). The Measurement of Emotional Intelligence:A Critical Review of the Literature and Recommendations for Researchers and Practitioners. Front Psychol.

[15] Cruess R, McIlroy JH, Cruess S, Ginsburg S, Steinert Y (2006). The professionalism mini-evaluation exercise:a preliminary investigation. Acad Med.

[16] Partido BB, Stefanik D, Rashid W (2021). Relationship between emotional intelligence and professionalism among second-year dental students. J Dent Educ.

[17] Karukivi M, Kortekangas-Savolainen O, Saxén U, Haapasalo-Pesu K-M (2015). Professionalism Mini-Evaluation Exercise in Finland:a preliminary investigation introducing the Finnish version of the P-MEX instrument. J Adv Med Educ Prof.

[18] Farah-Franco S, Singer-Chang G, Deoghare H (2017). Advancing the measurement of dental students'professionalism. J Dent Educ.

[19] Sobani Z-u-A, Mohyuddin MM, Saeed SA, Farooq F, Qaiser KN, Gani F (2013). Professionalism in medical students at a private medical college in Karachi, Pakistan. J Pak Med Assoc.

[20] Bhutto SN, Asif M, Jawaid M (2015). Professionalism among medical students at two public sector universities-a comparative study. J Postgrad Med Inst.

[21] Imran N, Aftab MA, Haider II, Farhat A (2013). Educating tomorrow's doctors:A cross sectional survey of emotional intelligence and empathy in medical students of Lahore. Pak J Med Sci.

[22] Vasefi A, Dehghani M, Mirzaaghapoor M (2018). Emotional intelligence of medical students of Shiraz University of Medical Sciences cross sectional study. Ann Med Surg.

[23] Abe K, Niwa M, Fujisaki K, Suzuki Y (2018). Associations between emotional intelligence, empathy and personality in Japanese medical students. BMC Med Educ.

[24] Ahmed F, Mehak A, Ali S, Khan A, Shehzad S, Baloch Q (2017). The effect of emotional intelligence on academic performance of medical undergraduates. Int J Educ Psychol Res.

[25] McNulty JP, Mackay SJ, Lewis SJ, Lane S, White P (2016). An international study of emotional intelligence in first year radiography students:The relationship to age, gender and culture. Radiography.

[26] Haque M, Zulkifli Z, Haque SZ, Kamal ZM, Salam A, Bhagat V (2016). Professionalism perspectives among medical students of a novel medical graduate school in Malaysia. Adv Med Educ Pract.

[27] Govindaraja C, Ramachandran G, Min AKK (2016). The Ecology of Medical Professionalism:Perceived and Emulated, What matters?. MedEdPublish.

[28] Toriello HV, De Ridder V, Monica J, Brewer P, Mavis B, Allen R Emotional intelligence in undergraduate medical students:a scoping review. Adv Health Sci Educ.

[29] Mahaur R, Jain P, Jain AK (2017). Association of mental health to emotional intelligence in medical undergraduate students:are there gender differences. Indian J Physiol Pharmacol.

[30] Gorgich EAC, Barfroshan S, Ghoreishi G, Balouchi A, Nastizaie N, Arbabisarjou A (2016). The association of self-assessed emotional intelligence with academic achievement and general health among students of medical sciences. Glob J Health Sci.

[31] Ibrahim NK, Algethmi WA, Binshihon SM, Almahyawi RA, Alahmadi RF, Baabdullah MY (2017). Predictors and correlations of emotional intelligence among medical students at King Abdulaziz University, Jeddah. Pak J Med Sci.

[32] Reed S, Kassis K, Nagel R, Verbeck N, Mahan JD, Shell R (2015). Does emotional intelligence predict breaking bad news skills in pediatric interns?A pilot study. Med Educ Online.

[33] Humphrey-Murto S, Leddy JJ, Wood TJ, Puddester D, Moineau G (2014). Does emotional intelligence at medical school admission predict future academic performance?. Acad Med.

